# Supplementary material for: Structural variation at the maize WUSCHEL1 locus alters stem cell organization in inflorescences
Source: Nat Commun. 2021 Apr 22;12:2378. doi: 10.1038/s41467-021-22699-8 (PMC8062686; doi:10.1038/s41467-021-22699-8)
Supplement: Supplementary file 1 — Supplementary information [file 41467_2021_22699_MOESM1_ESM.pdf]

SUPPLEMENTARY FIGURES

STRUCTURAL VARIATION AT THE MAIZE *WUSCHEL1* LOCUS ALTERS STEM CELL ORGANIZATION IN INFLORESCENCES

Zongliang Chen, Wei Li, Craig Gaines, Amy Buck, Mary Galli, Andrea Gallavotti

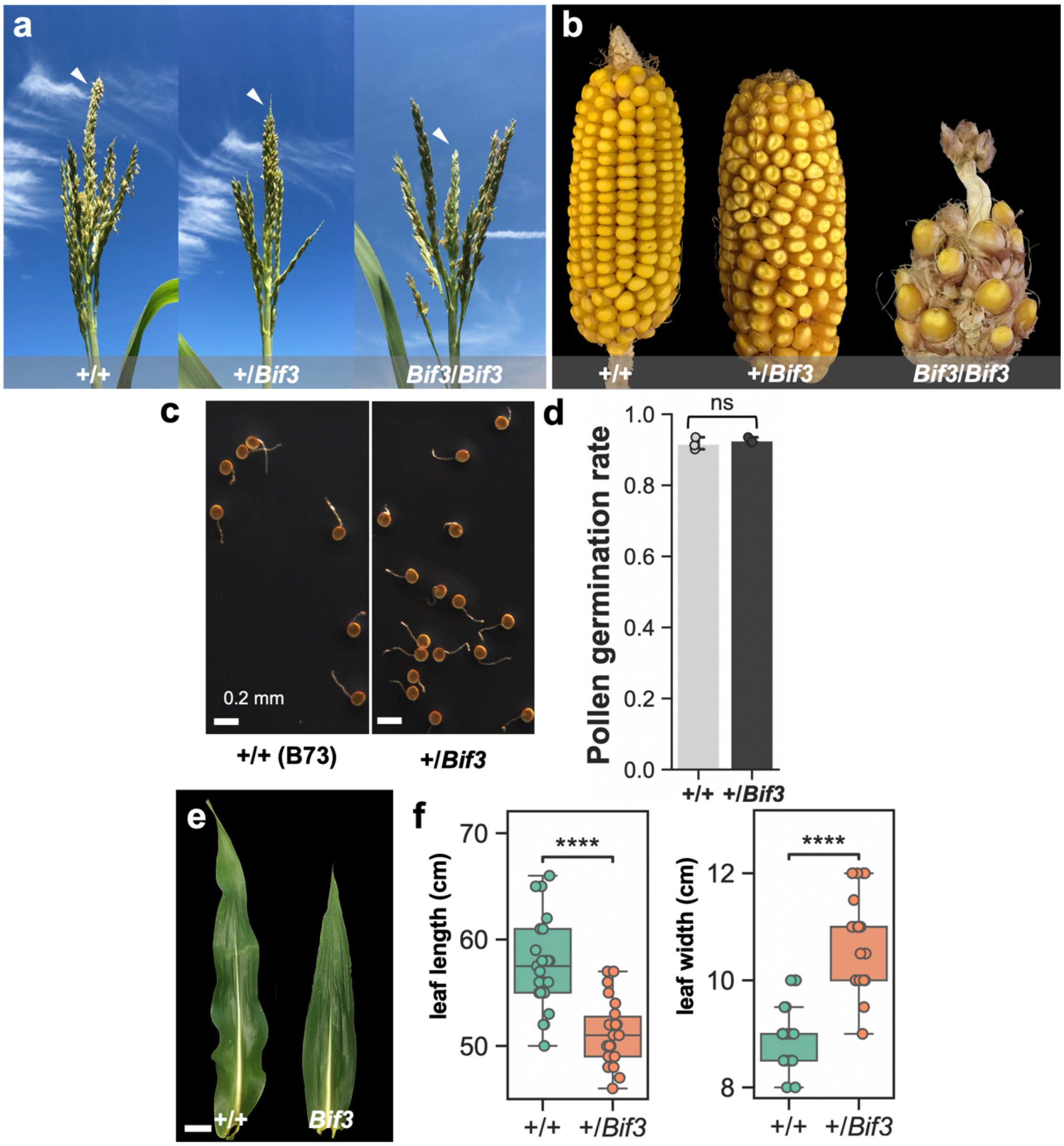

Supplementary Figure 1. *Bif3* mutant phenotype. (**a**) Mature tassel phenotype in B73. Normal tassels produce spikelets and flowers on branches and central spikes. Central spikes are reduced in *Bif3* homozygous mutants. White arrowheads point to the central spikes. (**b**) Mature ear phenotype in B73. Ears are reduced in size in *Bif3/Bif3* mutants and display disorganized rows of kernels in +/*Bif3* mutants. (**c,d**) Pollen germination in wild type and *+/Bif3* mutant in B73. Quantification of germination rate: 91.6% (3 plants, 10 anthers/plant, n=1400 pollen grains) in B73 and 92.6% (3 plants, 10 anthers/plant, n=4952 pollen grains) in *+/Bif3* (**d**); plotted data represent the mean, +/- standard deviation. (**e,f**) Leaves (the top third ones) from wild type and heterozygous *Bif3* plants in

A619. The *Bif3* leaves are broader in width and shorter in length (n_{WT} = 25, n_{Bif3} = 22, two-tailed Student's *t*-test, **** p<0.0001). Box plot center line, median; box limits, upper and lower quartiles; whiskers, maximum and minimum values. Single datapoints outside of the whiskers represent outliers. Scale bars: 1 cm in **b**, 200 µm in **c**; 5 cm in **e**.

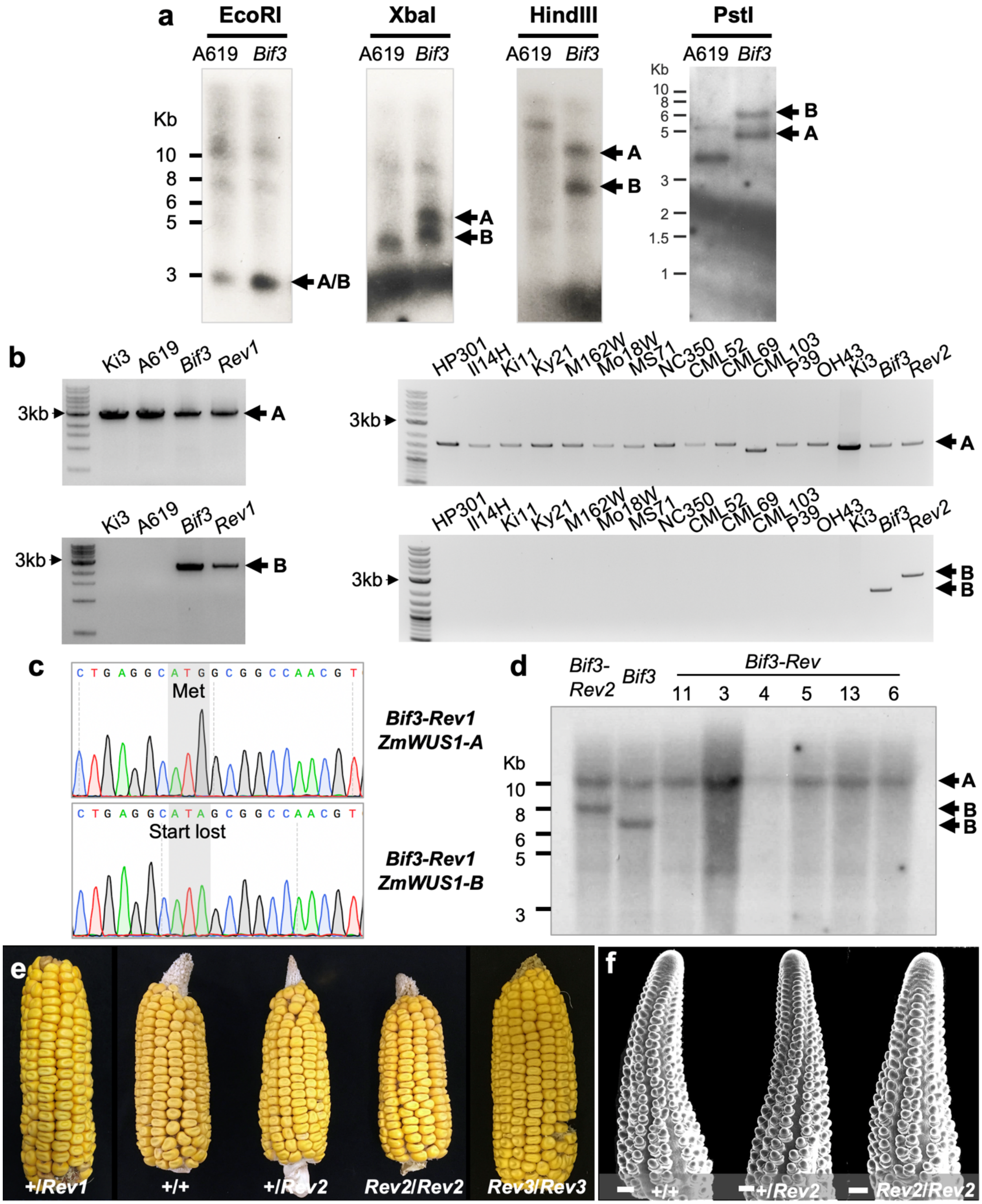

Supplementary Figure 2. Duplication of *ZmWUS1* in *Bif3*. (**a**) Southern blot analysis of *ZmWUS1* copies in wild type (A619) and *Bif3* mutants. A, *ZmWUS1-A*; B, *ZmWUS1-B*. (**b**) PCR amplification in different inbred lines of *ZmWUS1-A* and *ZmWUS1-B* by copy-

specific primers (*ZmWUS1-A,* 1428WUS-FWD/WUSseqR3; *ZmWUS1-B*, 6968F7/WUSseqR3 in left panels; *ZmWUS1-A,* 1428WUS-FWD/WUS-R; *ZmWUS1-B*, 6968F6/WUS-R in right panels). (**c**) Chromatograph snapshot of *ZmWUS1-A* and *ZmWUS1-B* start codons in *Bif3-Rev1*. (**d**) Southern blot analysis of *ZmWUS1*. (**e**) A normal ear phenotype is observed in *Rev1*, *Rev2* and *Rev3* plants. (**f**) SEM analysis of *Rev2* ear primordia, showing a regular arrangements of AMs at the IM flanks. Scale bars, 100μm.

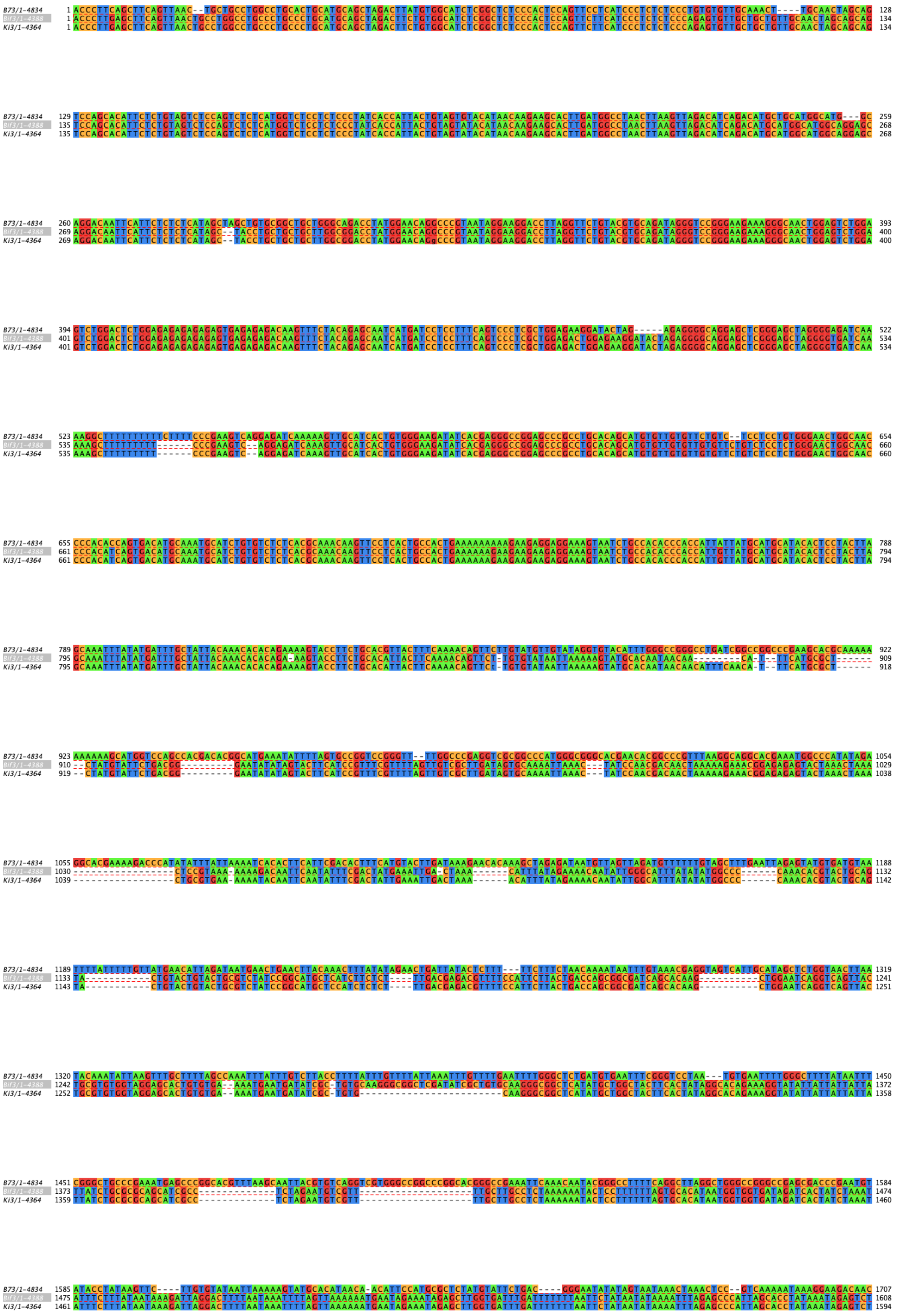
B73/1-4834
Ki3/1-4364

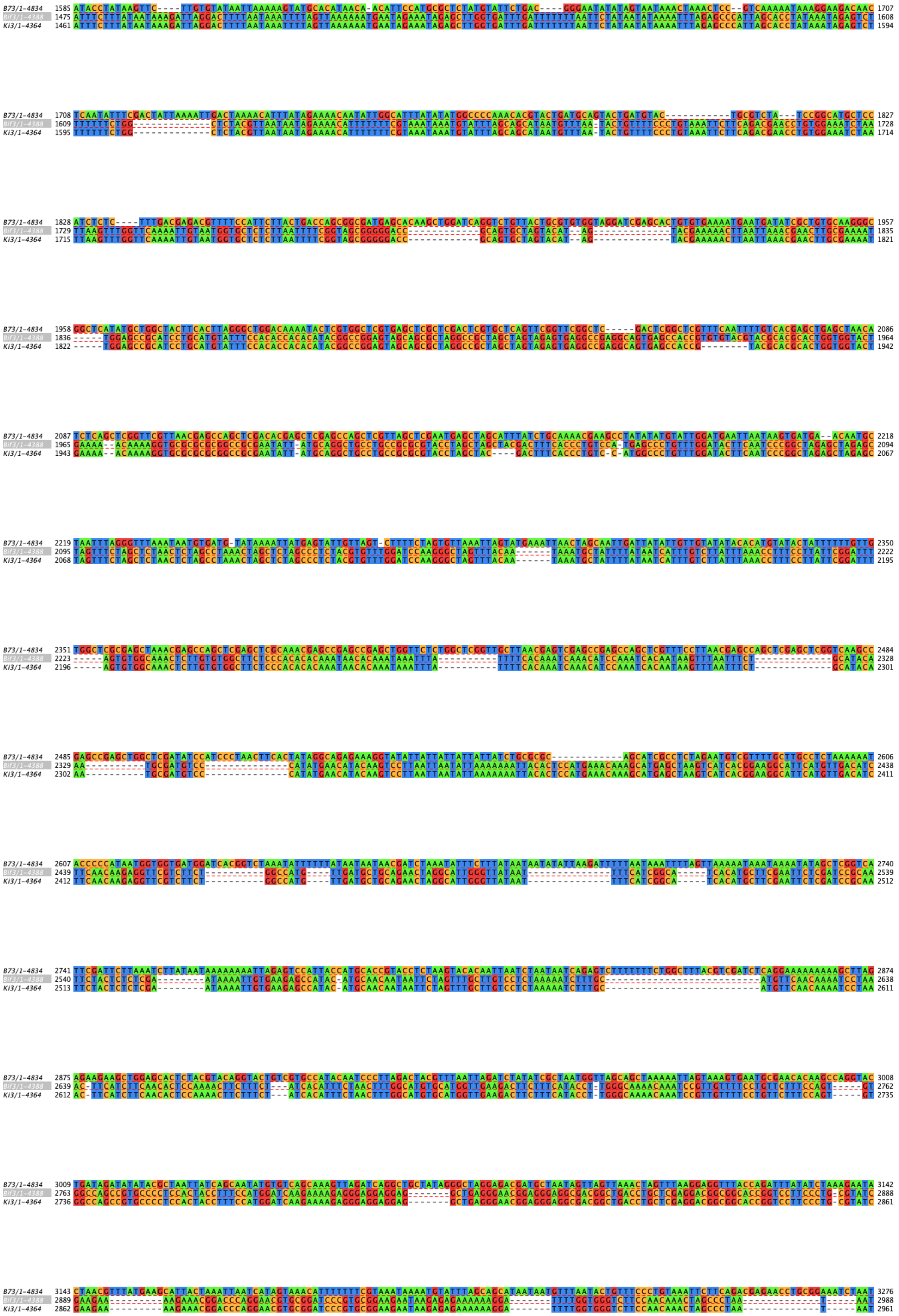

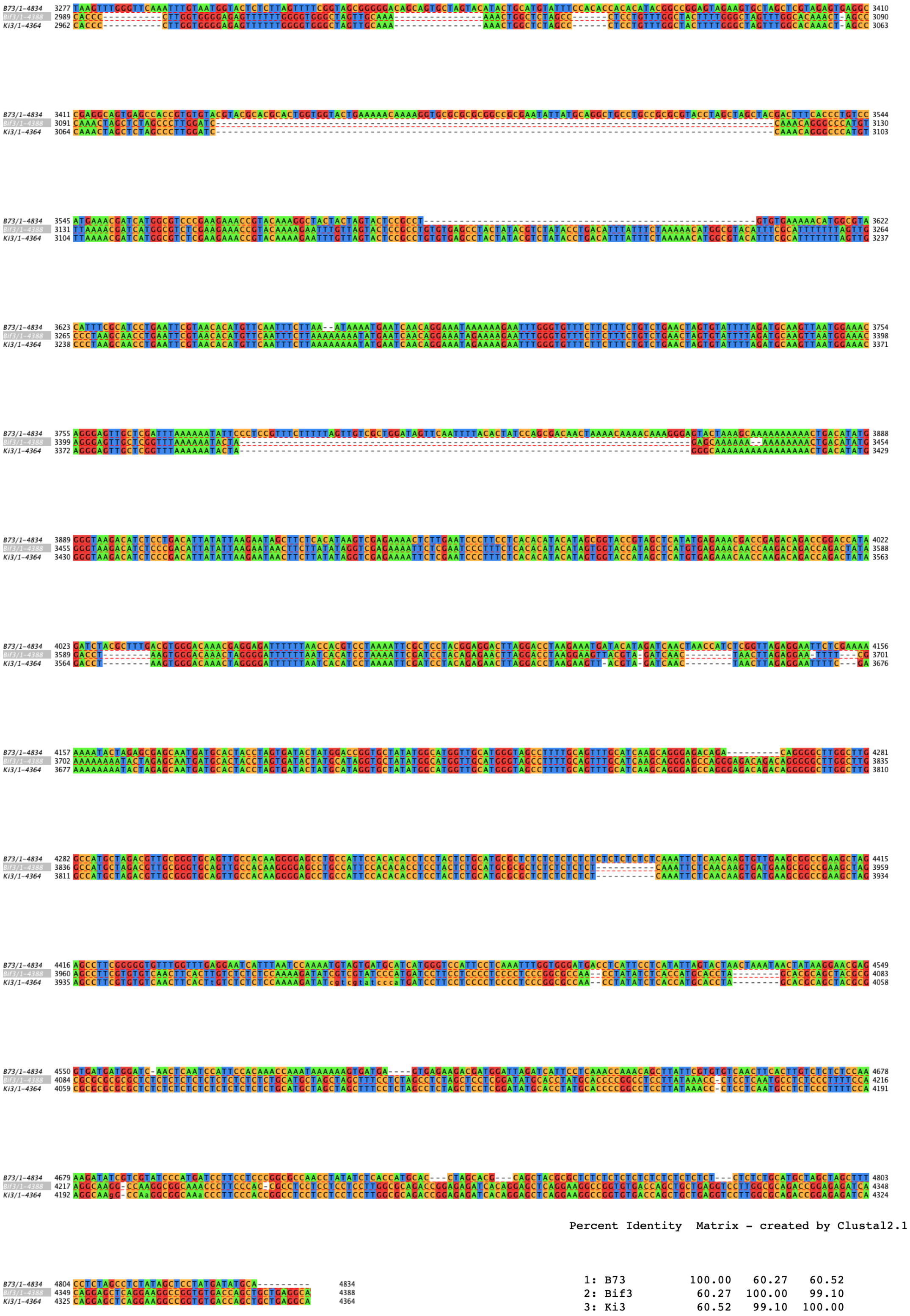

Supplementary Figure 3. Clustal Omega multiple sequence alignment of the *ZmWUS1*

promoter in *Bif3*, Ki3 and B73. Partial section (B73 region coordinates -4,978 to -144 from *ZmWUS1* start site). Alignment visualized in Jalview (version 2.11.1.3) [1].

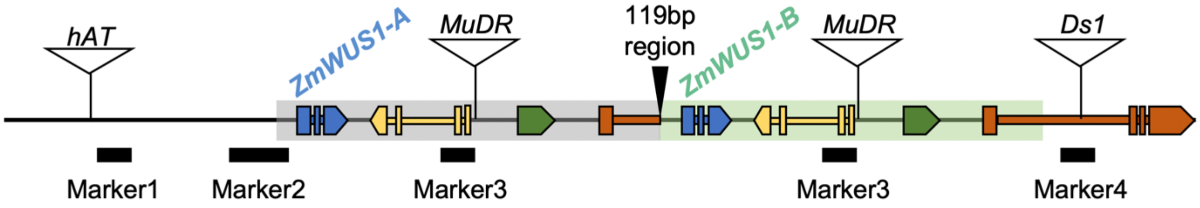

Revertant	Marker1	Marker2	*ZmWUS1-A*	Marker3	MuDR	*ZmWUS1-B*	Marker4	phenotype
Rev1	*Bif3/Bif3*	*Bif3/Bif3*	+	*Bif3/Bif3*	+	+ (G -> A)	*Bif3/Bif3*	WT
Rev2	*Bif3/Bif3*	*Bif3/Bif3*	+	*Bif3/Bif3*	+	+ (longer promoter)	*Bif3/Bif3*	WT
Rev3	*Bif3/Bif3*	*Bif3/Bif3*	+	*Bif3/Bif3*	+	-	*Bif3/Bif3*	WT
Rev4	*Bif3/Bif3*	*Bif3/Bif3*	+	*Bif3/Bif3*	+	-	*Bif3/Bif3*	WT
Rev5	*Bif3/Bif3*	*Bif3/Bif3*	+	*Bif3/Bif3*	+	-	*Bif3/Bif3*	WT
Rev6	*Bif3/Bif3*	*Bif3/Bif3*	+	*Bif3/Bif3*	+	-	*Bif3/Bif3*	WT
Rev7	*Bif3/Bif3*	*Bif3/Bif3*	+	*Bif3/Bif3*	+	-	*Bif3/Bif3*	WT
Rev8	*Bif3/Bif3*	*Bif3/Bif3*	+	*Bif3/Bif3*	+	-	*Bif3/Bif3*	WT
Rev9	*Bif3/Bif3*	*Bif3/Bif3*	+	*Bif3/Bif3*	+	-	*Bif3/Bif3*	WT
Rev10	*Bif3/Bif3*	*Bif3/Bif3*	+	*Bif3/Bif3*	+	-	*Bif3/Bif3*	WT
Rev11	*Bif3/Bif3*	*Bif3/Bif3*	+	*Bif3/Bif3*	+	-	*Bif3/Bif3*	WT
Rev12	*Bif3/Bif3*	*Bif3/Bif3*	+	*Bif3/Bif3*	+	-	*Bif3/Bif3*	WT
Rev13	*Bif3/Bif3*	*Bif3/Bif3*	+	*Bif3/Bif3*	+	-	*Bif3/Bif3*	WT
Rev14	*Bif3/Bif3*	*+/Bif3*	+	*+/Bif3*	+	-	*+/Bif3*	WT
Rev15	*Bif3/Bif3*	*Bif3/Bif3*	+	*Bif3/Bif3*	+	-	*Bif3/Bif3*	WT
Rev16	*Bif3/Bif3*	*Bif3/Bif3*	+	*Bif3/Bif3*	+	-	*Bif3/Bif3*	WT
Rev17	*Bif3/Bif3*	*Bif3/Bif3*	+	*Bif3/Bif3*	+	-	*Bif3/Bif3*	WT
Rev18	*Bif3/Bif3*	*Bif3/Bif3*	+	*Bif3/Bif3*	+	-	*Bif3/Bif3*	WT
Rev19	*Bif3/Bif3*	*Bif3/Bif3*	+	*Bif3/Bif3*	+	-	*Bif3/Bif3*	WT
Rev20	*Bif3/Bif3*	*Bif3/Bif3*	+	*Bif3/Bif3*	+	-	*Bif3/Bif3*	WT

Supplementary Figure 4. Genotyping of the *Bif3* locus in different revertant individuals. Each original M1 revertant was self-crossed and homozygous *Bif3/Bif3* plants were selected based on Marker 1 for genotyping with additional markers spanning the entire region. Details on the markers used are provided in Supplementary Data 2.

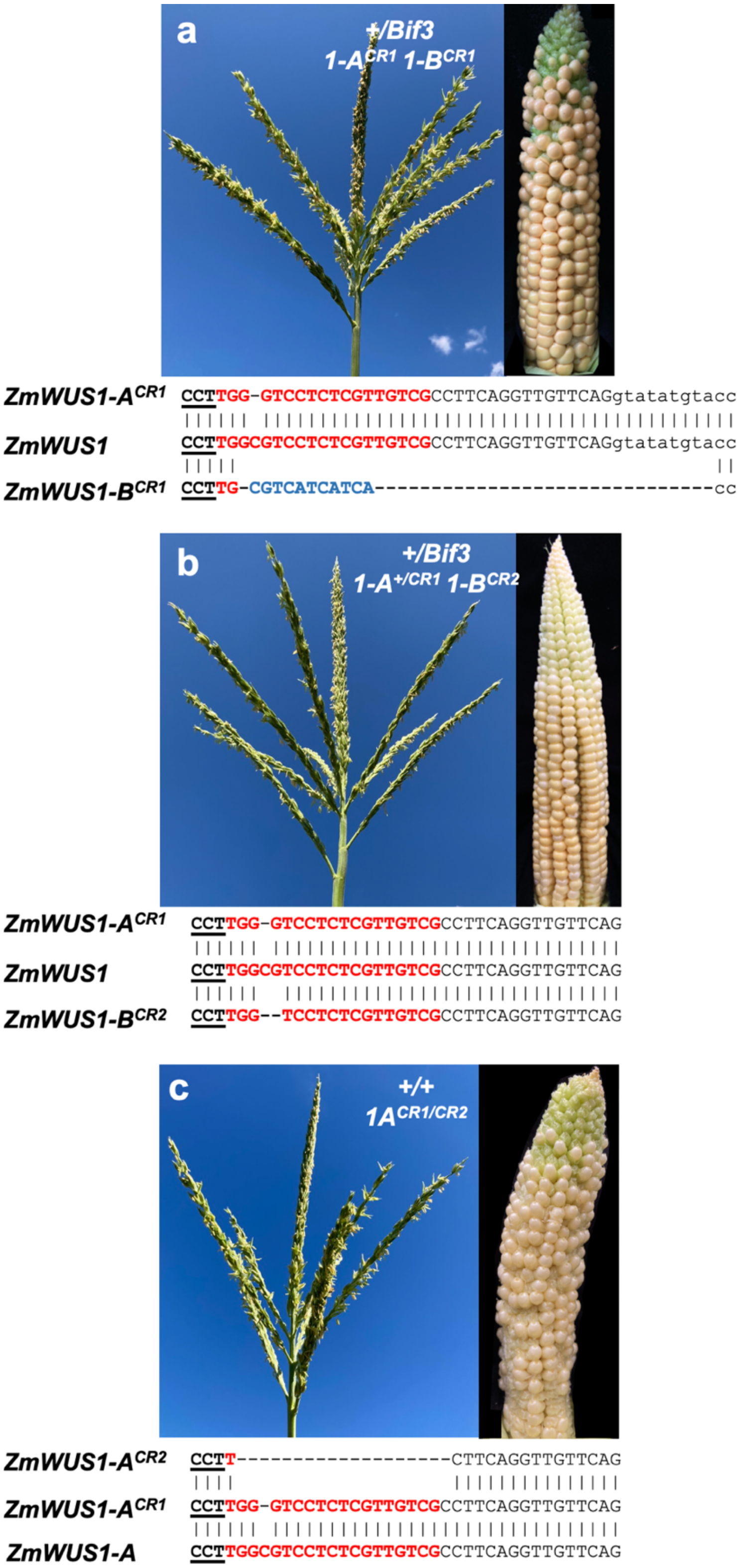

Supplementary Figure 5. CRISPR-Cas9 loss-of-function *ZmWUS1-A* and *ZmWUS1-B* alleles. Additional knockout events in both genes in *Bif3* mutant (**a**,**b**) and wild type (**c**) plants show a wild type phenotype in both tassels and ears. Events are homozygous, unless otherwise noted.

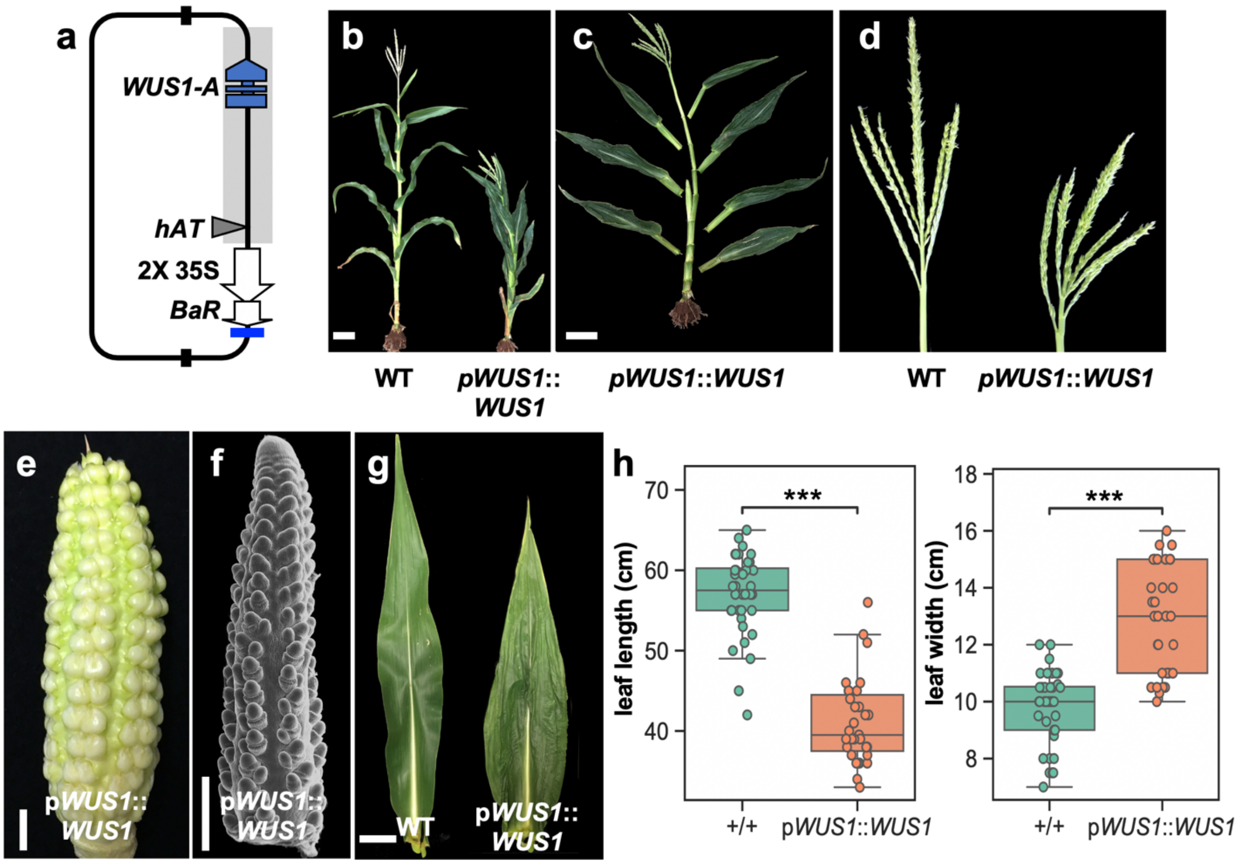

Supplementary Figure 6. Analysis of *pWUS1-A::ZmWUS1-A* transgenic plants. (**a**) Binary *pZmWUS1-A*::*ZmWUS1-A* construct used for transformation. (**b**,**c**) *pWUS1-A* transgenic plants are shorter and more compact. (**d**) Normal tassels of *pWUS1-A* transgenic plants. (**e**) Normal ear appearance of *pWUS1-A* transgenic plants. (**f**) SEM image of *pWUS1-A* transgenic ear primordia. (**g**,**h**) *pWUS1-A* transgenic plants have wider and shorter leaves (n_{WT} = 36, $n_{pWUS1\text{-}A}$ = 31, two-tailed Student's *t*-test, ***$p<0.001$). Box plot center line, median; box limits, upper and lower quartiles; whiskers, maximum and minimum values. Single datapoints outside of the whiskers represent outliers. Bars: 10cm in **b** and **c**, 1cm in **e**, 500μm in **f**, 5cm in **g**.

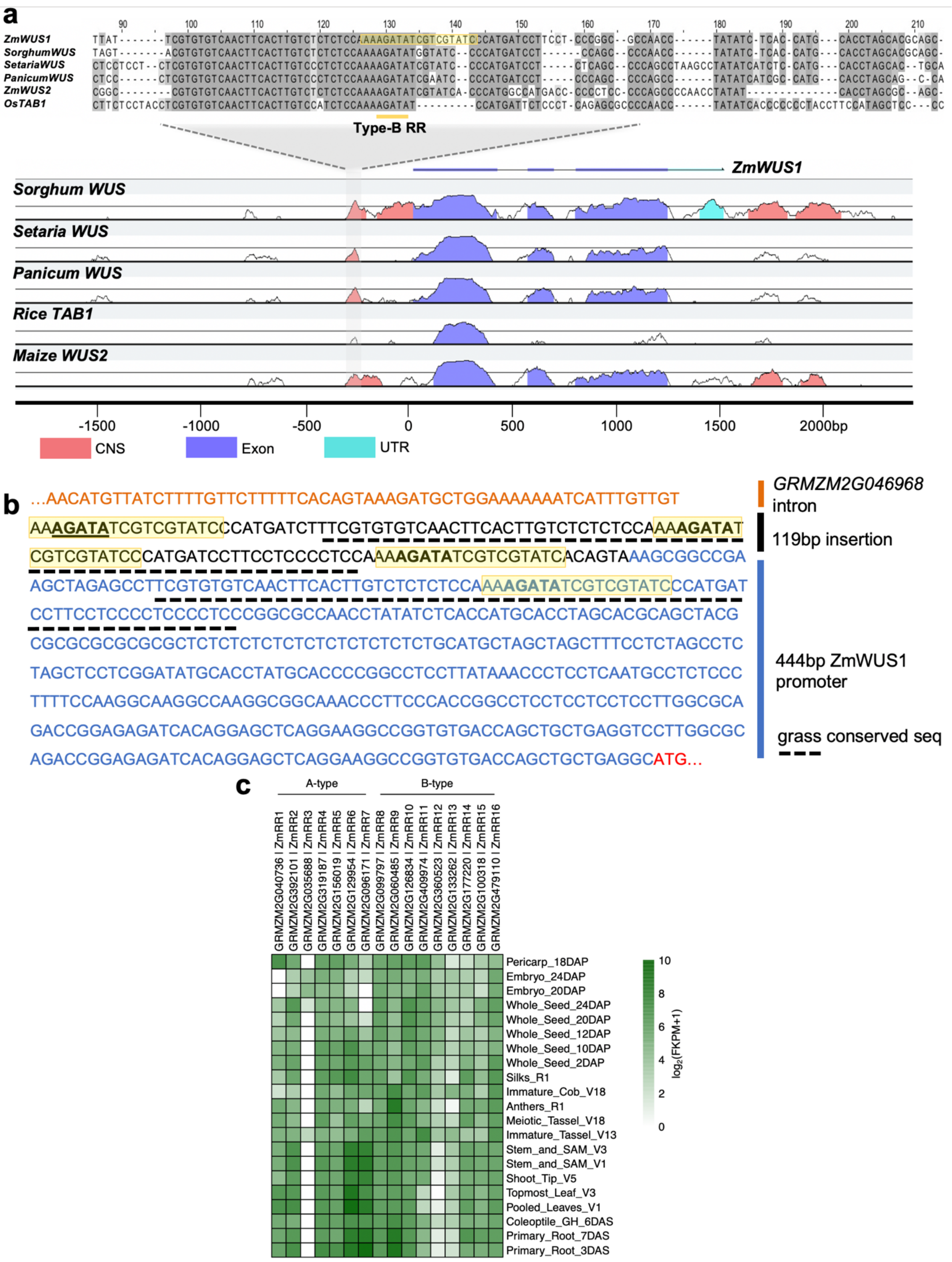
a
ZmWUS1
SorghumWUS
SetariaWUS
PanicumWUS
ZmWUS2
OsTAB1
Type-B RR
ZmWUS1
Sorghum WUS
Setaria WUS
Panicum WUS
Rice TAB1
Maize WUS2
-1500
-1000
-500
0
500
1000
1500
2000bp
CNS
Exon
UTR
b
GRMZM2G046968 intron
119bp insertion
444bp ZmWUS1 promoter
grass conserved seq
c
A-type
B-type
log2(FKPM+1)
Pericarp_18DAP
Embryo_24DAP
Embryo_20DAP
Whole_Seed_24DAP
Whole_Seed_20DAP
Whole_Seed_12DAP
Whole_Seed_10DAP
Whole_Seed_2DAP
Silks_R1
Immature_Cob_V18
Anthers_R1
Meiotic_Tassel_V18
Immature_Tassel_V13
Stem_and_SAM_V3
Stem_and_SAM_V1
Shoot_Tip_V5
Topmost_Leaf_V3
Pooled_Leaves_V1
Coleoptile_GH_6DAS
Primary_Root_7DAS
Primary_Root_3DAS

Supplementary Figure 7. Conservation of type-B RR binding sites in the promoter of *WUS* in different species. (**a**) Alignment of the conserved region in the proximal promoter region of the *WUS1* gene in different grass species. In yellow is the 17bp element repeated in the unique 119bp region of the *Bif3* mutant. (**b**) Proximal promoter sequence of the *ZmWUS1-B* gene in *Bif3* mutants. (**c**) RNA-seq expression levels of *ZmRR* genes in different tissues, from Stelpflug et al. 2016 [2].

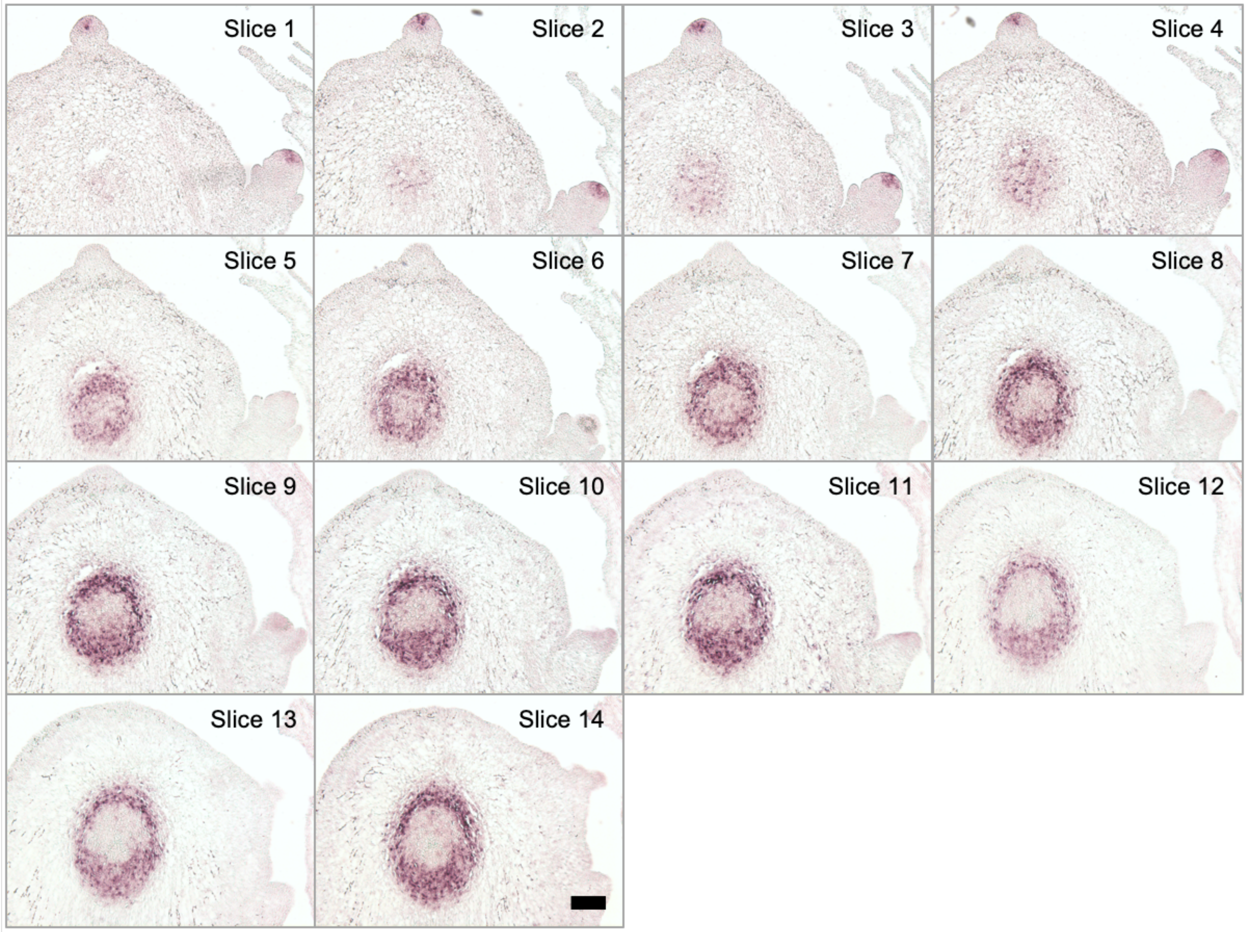

Supplementary Figure 8. RNA *in situ* hybridizations. *ZmWUS1* antisense probe used on consecutive longitudinal sections of ear primordia show a hollow spherical expression domain in *Bif3* inflorescence meristem (A619 background). Bar: 100 μm.

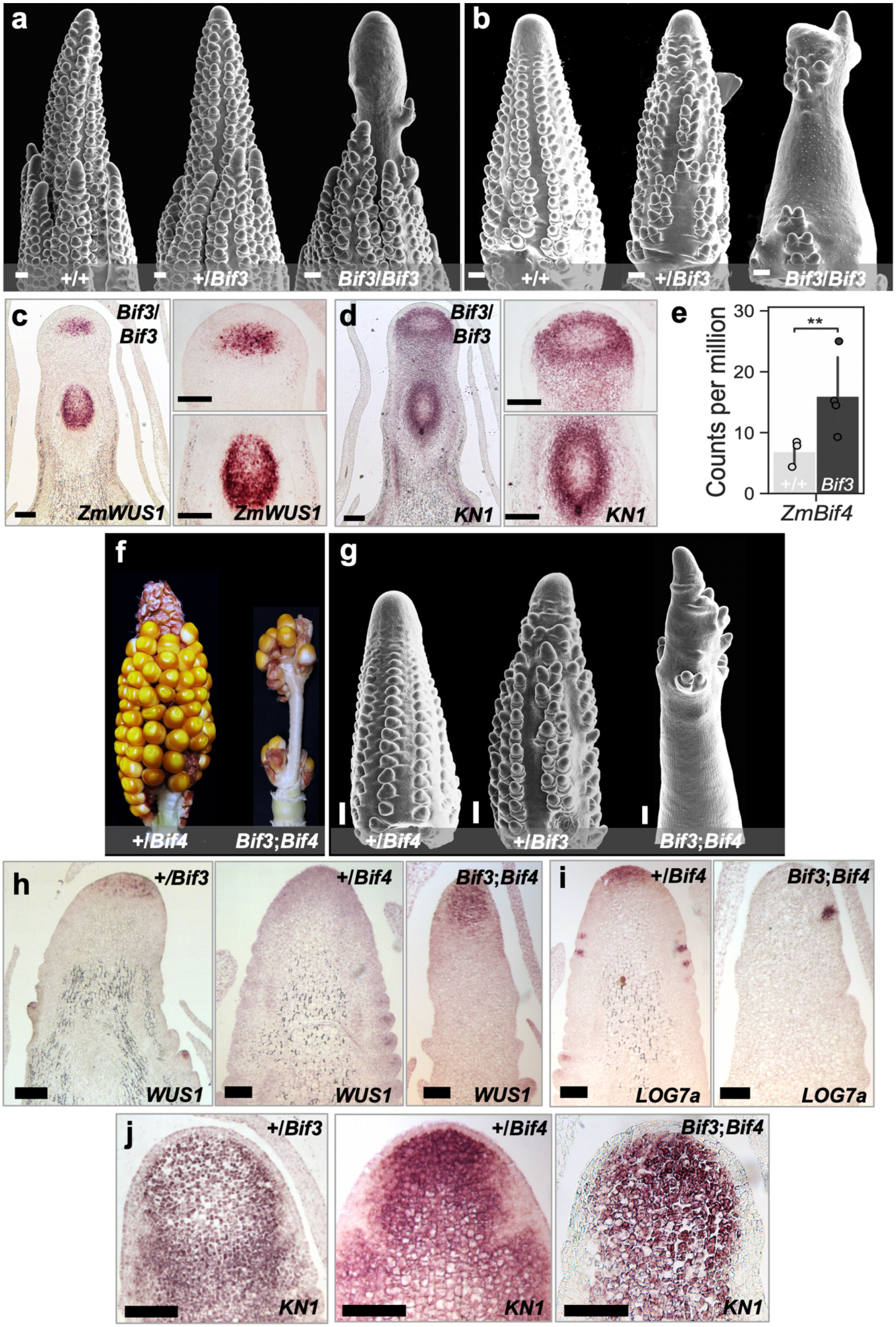
a
+/+
+/Bif3
Bif3/Bif3
b
+/+
+/Bif3
Bif3/Bif3
c
Bif3/
Bif3
ZmWUS1
ZmWUS1
d
Bif3/
Bif3
KN1
KN1
e
Counts per million
30
20
10
0
**
+/+
Bif3
ZmBif4
f
+/Bif4
Bif3;Bif4
g
+/Bif4
+/Bif3
Bif3;Bif4
h
+/Bif3
+/Bif4
Bif3;Bif4
WUS1
WUS1
WUS1
i
+/Bif4
Bif3;Bif4
LOG7a
LOG7a
j
+/Bif3
+/Bif4
Bif3;Bif4
KN1
KN1
KN1

Supplementary Figure 9. *Bif3* mutant phenotype in the B73 background and genetic interaction between *Bif3* and *Bif4* mutants. (**a**,**b**) SEMs of wild-type and *Bif3* tassel (**a**) and ear (**b**) primordia. *Bif3* inflorescences shows reduced number of SPMs and single SMs. (**c**,**d**) mRNA *in situ* hybridizations of *ZmWUS1* (**c**) and *KN1* (**d**) in *Bif3/Bif3* inflorescence meristems show two OC domains. (**e**) The *BIF4* gene is upregulated about 1.7-fold in *Bif3* ear primordia; two-tailed Student's *t*-test **p<0.01; plotted data represent the mean, +/- standard deviation; $n_{+/+}$=3, $n_{Bif3/Bif3}$=4 (*n* pools of three ear samples). (**f**,**g**) Enhanced *Bif3* ear phenotype in heterozygous *Bif3*;*Bif4* double mutants. (**h**-**j**) Expression pattern of *ZmWUS1*, *ZmLOG7a* and *KN1* in *Bif3* heterozygous and *Bif3*;*Bif4* double mutants. Scale bars: 200 μm in **a**, **b**, **g;** 100 μm in **c**, **d**, **h**, **i**.

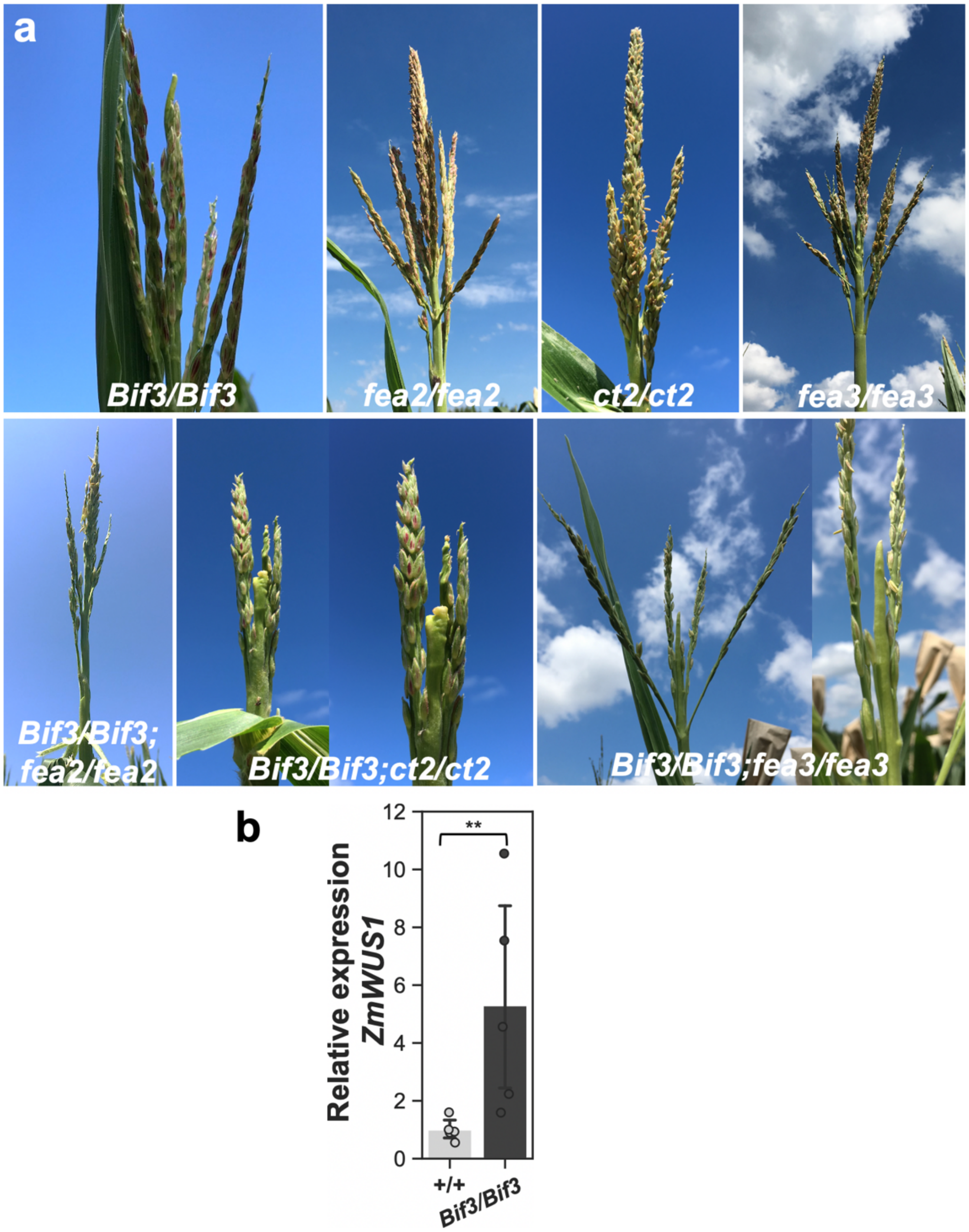

Supplementary Figure 10. Double mutant analysis. Mature tassel phenotype in single and double mutant combinations of *Bif3* and different fasciated mutants. The central spike is particularly reduced in length in *Bif3/Bif3;ct2/ct2* and *Bif3/Bif3;fea3/fea3* mutants (n≥8) (**b**) qRT-PCR showing *ZmWUS1* overexpression in developing embryos 12 days after pollination. Two-tailed Student's t-test **p<0.01; plotted data represent the mean, +/- standard deviation; $n_{+/+}$=5, $n_{Bif3/Bif3}$=5 (*n* pools of three ear samples).

REFERENCES

1. Waterhouse, A.M. *et al.* Jalview Version 2--a multiple sequence alignment editor and analysis workbench. *Bioinformatics* **25**, 1189-91 (2009).
2. Stelpflug, S.C. *et al.* An Expanded Maize Gene Expression Atlas based on RNA Sequencing and its Use to Explore Root Development. *Plant Genome* **9**(2016).
