# Supplementary material for: Structural variation at the maize WUSCHEL1 locus alters stem cell organization in inflorescences
Source: Nat Commun. 2021 Apr 22;12:2378. doi: 10.1038/s41467-021-22699-8 (PMC8062686; doi:10.1038/s41467-021-22699-8)
Supplement: Supplementary file 3 — Descriptions of Additional Supplementary Files [file 41467_2021_22699_MOESM3_ESM.pdf]

Descriptions of Additional Supplementary Files

Supplementary Data 1
Description: List of DEGs by RNA-seq analysis of Bif3 ear primordia.

Supplementary Data 2
Description: List of molecular markers, primers and in situ probes used in this study.
