# Supplementary material for: Structural variation at the maize WUSCHEL1 locus alters stem cell organization in inflorescences
Source: Nat Commun. 2021 Apr 22;12:2378. doi: 10.1038/s41467-021-22699-8 (PMC8062686; doi:10.1038/s41467-021-22699-8)
Supplement: Supplementary file 6 — Reporting summary [file 41467_2021_22699_MOESM6_ESM.pdf]

nature research

Corresponding author(s): Andrea Gallavotti

Last updated by author(s): Mar 19, 2021

Reporting Summary

Nature Research wishes to improve the reproducibility of the work that we publish. This form provides structure for consistency and transparency in reporting. For further information on Nature Research policies, see our Editorial Policies and the Editorial Policy Checklist.

Statistics

For all statistical analyses, confirm that the following items are present in the figure legend, table legend, main text, or Methods section.

n/a	Confirmed	
☐	☒	The exact sample size (n) for each experimental group/condition, given as a discrete number and unit of measurement
☐	☒	A statement on whether measurements were taken from distinct samples or whether the same sample was measured repeatedly
☐	☒	The statistical test(s) used AND whether they are one- or two-sided<br>*Only common tests should be described solely by name; describe more complex techniques in the Methods section.*
☒	☐	A description of all covariates tested
☒	☐	A description of any assumptions or corrections, such as tests of normality and adjustment for multiple comparisons
☐	☒	A full description of the statistical parameters including central tendency (e.g. means) or other basic estimates (e.g. regression coefficient) AND variation (e.g. standard deviation) or associated estimates of uncertainty (e.g. confidence intervals)
☐	☒	For null hypothesis testing, the test statistic (e.g. F, t, r) with confidence intervals, effect sizes, degrees of freedom and P value noted<br>*Give P values as exact values whenever suitable.*
☒	☐	For Bayesian analysis, information on the choice of priors and Markov chain Monte Carlo settings
☒	☐	For hierarchical and complex designs, identification of the appropriate level for tests and full reporting of outcomes
☒	☐	Estimates of effect sizes (e.g. Cohen's d, Pearson's r), indicating how they were calculated

Our web collection on statistics for biologists contains articles on many of the points above.

Software and code

Policy information about availability of computer code

Data collection: Gene expression differences were quantified using the Eco Real-Time PCR System Software v4 (Illumina).

Data analysis: RNA-seq raw data were trimmed with Trimmomatic with default settings63 and aligned to the reference genome (B73v3 genome) using hisat 2.1.0 with default settings. Gene expression was quantified as read counts by htseq-count and genes with differential expressions were determined by edgeR 3.18.1 package. All statistical analyses of gene expression were conducted in R and python. Genes with a fold change >1.5 with p<0.05 and FDR<0.1 were considered as differentially expressed genes. IGV was used for genomic data visualization. The software Ugene was used to map ZmWUS1 sequence reads.

For manuscripts utilizing custom algorithms or software that are central to the research but not yet described in published literature, software must be made available to editors and reviewers. We strongly encourage code deposition in a community repository (e.g. GitHub). See the Nature Research guidelines for submitting code & software for further information.

Data

Policy information about availability of data

All manuscripts must include a data availability statement. This statement should provide the following information, where applicable:

- Accession codes, unique identifiers, or web links for publicly available datasets
- A list of figures that have associated raw data
- A description of any restrictions on data availability

RNA-seq datasets are deposited in GEO (GSE158330). Genomic sequences of ZmWUS1-A and ZmWUS1-B loci in the Bif3 mutant are available in GenBank (accession numbers MW677561, MW677562, MW677563). Source data are provided with this paper.

Field-specific reporting

Please select the one below that is the best fit for your research. If you are not sure, read the appropriate sections before making your selection.

☒ Life sciences ☐ Behavioural & social sciences ☐ Ecological, evolutionary & environmental sciences

For a reference copy of the document with all sections, see nature.com/documents/nr-reporting-summary-flat.pdf

Life sciences study design

All studies must disclose on these points even when the disclosure is negative.

Sample size	No sample-size calculations was performed. Sample size was determined to be sufficient based on obvious statistically significant differences.
Data exclusions	No data was excluded from analysis
Replication	All experiments were replicated at least two times and/or in multiple biological samples with the same outcome.
Randomization	No strict randomization was used. In each experiment, samples were genotyped or phenotyped and assigned to a specific homogeneous group, often for pooling purposes that required genotype information. Samples were grown in a randomized fashion in the field, ensuring that the phenotype characterization was randomized.
Blinding	In general, no blinding was used because of the required genotype information and/or the obvious phenotype. e.g. for phenotypic measurements the mutant phenotype is obvious. However, genotype and phenotype information was matched only after analysis when possible.

Reporting for specific materials, systems and methods

We require information from authors about some types of materials, experimental systems and methods used in many studies. Here, indicate whether each material, system or method listed is relevant to your study. If you are not sure if a list item applies to your research, read the appropriate section before selecting a response.

Materials & experimental systems

n/a	Involved in the study	
☒	☐	Antibodies
☒	☐	Eukaryotic cell lines
☒	☐	Palaeontology and archaeology
☒	☐	Animals and other organisms
☒	☐	Human research participants
☒	☐	Clinical data
☒	☐	Dual use research of concern

Methods

n/a	Involved in the study	
☒	☐	ChIP-seq
☒	☐	Flow cytometry
☒	☐	MRI-based neuroimaging
